# Antioxidant Alternatives in the Treatment of Amyotrophic Lateral Sclerosis: A Comprehensive Review

**DOI:** 10.3389/fphys.2020.00063

**Published:** 2020-02-06

**Authors:** Sandra Carrera-Juliá, Mari Luz Moreno, Carlos Barrios, Jose Enrique de la Rubia Ortí, Eraci Drehmer

**Affiliations:** ^1^Doctoral Degree’s School, Catholic University of Valencia “San Vicente Mártir”, Valencia, Spain; ^2^Department of Nutrition and Dietetics, Catholic University of Valencia “San Vicente Mártir”, Valencia, Spain; ^3^Department of Basic Sciences, Catholic University of Valencia “San Vicente Mártir”, Valencia, Spain; ^4^Institute for Research on Musculoskeletal Disorders, Catholic University of Valencia “San Vicente Mártir”, Valencia, Spain; ^5^Department of Basic Medical Sciences, Catholic University of Valencia “San Vicente Mártir”, Valencia, Spain

**Keywords:** amyotrophic lateral sclerosis, neurodegenerative diseases, oxidative stress, mitochondrial dysfunction, nicotinamide riboside, pterostilbene

## Abstract

Amyotrophic lateral sclerosis (ALS) is a neurodegenerative disease that produces a selective loss of the motor neurons of the spinal cord, brain stem and motor cortex. Oxidative stress (OS) associated with mitochondrial dysfunction and the deterioration of the electron transport chain has been shown to be a factor that contributes to neurodegeneration and plays a potential role in the pathogenesis of ALS. The regions of the central nervous system affected have high levels of reactive oxygen species (ROS) and reduced antioxidant defenses. Scientific studies propose treatment with antioxidants to combat the characteristic OS and the regeneration of nicotinamide adenine dinucleotide (NAD^+^) levels by the use of precursors. This review examines the possible roles of nicotinamide riboside and pterostilbene as therapeutic strategies in ALS.

## Oxidative Stress

Most eukaryotic organisms need oxygen to ensure the normal functioning of cellular energy metabolism, which is an evolutionary advantage and a highly efficient form of energy production (Jie et al., 2013). In the electron transport chain (ETC), oxygen is partially reduced by the incorporation of an electron, generating a free radical as a secondary product (Falkowski and Godfrey, 2008; Venditti et al., 2013). Free radicals are atoms or molecules that have one or more unpaired electrons in their last orbital layer (Chandrasekaran et al., 2017), which makes them strongly reactive capable of carrying out chain reactions, responsible for the oxidative damage of cells and tissues (Solleiro-Villavicencio and Rivas-Arancibia, 2018). They are classified as ROS and reactive nitrogen species (RNS) (De Gara and Foyer, 2017). Also included are reactive iron species (RIS) (Dixon and Stockwell, 2014) and copper species (RCS) (Gyulkhandanyan et al., 2003). The main ROS are: superoxide anion radicals (O${}_{2}^{∙-}$), hydroxyl radicals (OH^∙^) and hydrogen peroxide (H_2_O_2_) (Popa-Wagner et al., 2013). The RNS include: nitric oxide radical (NO^∙^), nitroxyl anion (NO^–^), nitrosonium cation (NO^+^) and peroxynitrite anions (ONOO^–^) (Halliwell, 2012; Rogers et al., 2014; Zuo et al., 2015).

**FIGURE 1:**
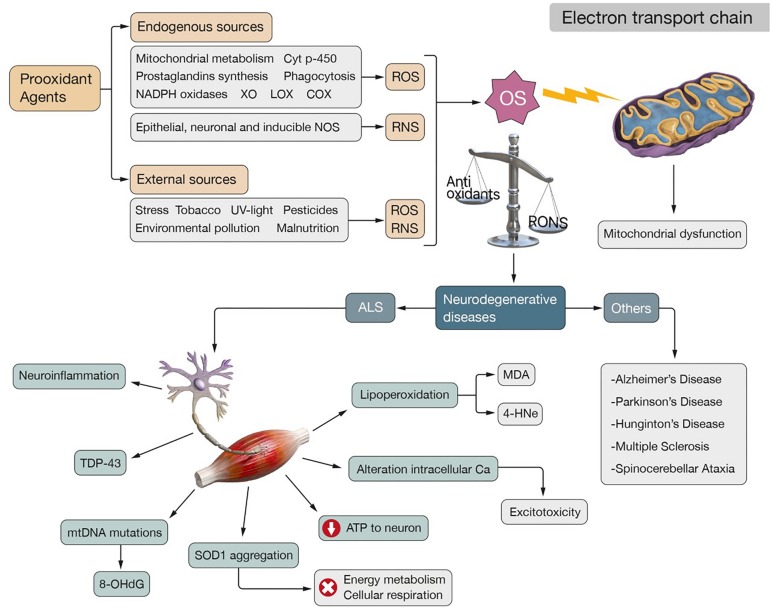
Physiopathogenesis of ALS. Oxidative stress is caused by an imbalance between antioxidant defenses and RONS in favor of these second ones. The endogenous production of ROS is secondary to different prooxidant agents: mitochondrial and cytochrome p-450 metabolism, prostaglandins synthesis, phagocytosis, NADPH oxidases, XO, LOX and COX. Among the endogenous sources of RNS it should be noted the activity of epithelial, neuronal and inducible NOS. Exogenous sources also can produce RONS: stress, tobacco, UV-light, pesticides, environmental pollution and malnutrition. OS affects the normal functioning of the ETC, producing mitochondrial dysfunction that generates more ROS, which leads to a “vicious-cycle” that increases metabolic stress. This situation characterizes neurodegenerative diseases such as: ALS, AD, PD, MS, HD, and SA. In the case of ALS, OS and mitochondrial dysfunction have been identified as two mechanisms involved in its pathogenesis. They are related to neuroinflammation, aggregation of TDP-43, mutations in the mtDNA that produce characteristic OS biomarkers such as 8-OHdG, aggregation of SOD1 which impairs energy metabolism and cellular respiration, affectation of ATP supply to neurons and disturbance of the intracellular calcium homeostasis that results in excitotoxicity. The formation of highly reactive end products such as MDA and 4-HNE, both secondary to the lipoperoxidation of the neuronal membranes, is also observed.

The production of ROS is mainly secondary to enzymatic reactions involved in the respiratory chain, activity of cytochrome p-450, synthesis of prostaglandins and phagocytosis (Pizzino et al., 2017). Mitochondrial activity and metabolism of cytochrome p-450 are the most contributing sources in mammalian cells (Jha et al., 2017) (Figure 1).

Mitochondrial production of ROS may occur on the external membrane, internal membrane or in the mitochondrial matrix, during physiological and pathological conditions (Pizzino et al., 2017). O${}_{2}^{∙-}$ production occurs when there is a buildup of nicotinamide adenine dinucleotide phosphate (NADPH) or when there is a reduced CoQ pool within the mitochondria (Murphy, 2009). O${}_{2}^{∙-}$ may also be secondary to the enzymatic activity of lipoxygenases (LOX) and cyclooxygenases (COX) (Valko et al., 2007) during the arachidonic acid metabolism and by endothelial and inflammatory cells (Al-Gubory et al., 2012). O${}_{2}^{∙-}$ may participate in reactions that produce H_2_O_2_ or OH^∙^ (Kumar and Pandey, 2015) (Figure 1).

The cytochrome p-450 enzymes present in the liver are an important source of ROS production and their function is to catalyze O${}_{2}^{∙-}$ producing reactions by NADPH dependent mechanisms (Liochev, 2013). The risk of ROS production here is high because it contains transition ions, oxygen and electron transfer processes (Liochev, 2013). In addition, there are a group of NOX (NADPH oxidases) enzymes located on the cell membrane of polymorphonuclear cells, macrophages and endothelial cells, that facilitate the conversion of oxygen into superoxide on biological membranes using NADPH as an electron donor with ROS released as secondary products (Atashi et al., 2015) (Figure 1). Endothelium xanthine dehydrogenase interacts with xanthine oxidase (XO) producing O${}_{2}^{∙-}$ and H_2_O_2_, and thus, generating another source of free radicals (Turrens, 2003) (Figure 1).

Non-enzymatic reactions may also be responsible for the production of ROS by the reaction of oxygen with organic compounds and after cellular exposure to ionizing radiation (Valko et al., 2007) (Figure 1).

The endogenous release of RNS, such as nitric oxide (NO^∙^), is produced from L-arginine in reactions of catalyze by three main isoforms of nitric oxide synthase (NOS): epithelial NOS, neuronal NOS and inducible NOS, which are activated in response to various endotoxin or cytokine signals (Förstermann and Sessa, 2012). Thus oxygen can react with this NO^∙^ and form highly reactive molecules such as ONOO^–^ (Salisbury and Bronas, 2015; Sharina and Martin, 2017) (Figure 1).

Endogenous production of reactive oxygen and nitrogen species (RONS) can be conditioned by exogenous pro-oxidant factors: environmental and atmospheric pollution, water pollution, chemicals like pesticides or industrial solvents, heavy metals or transition metals, different types of xenobiotics, irradiation by UV-light, X-rays or gamma rays, stress, tobacco, smoked meat, the use of waste oil and malnutrition (Phaniendra et al., 2015; Niedzielska et al., 2016; Solleiro-Villavicencio and Rivas-Arancibia, 2018; Zewen et al., 2018) (Figure 1).

Reactive oxygen and nitrogen species at physiological concentrations are regulators of numerous cellular functions: cellular signaling pathway, control of cell survival, regulation of vascular tone, signal transduction by cell membrane receptors, membrane renewal, synthesis and release of hormones, increase of inflammatory cytokine transcription regulation of the immune system (Robberecht, 2000; Ray et al., 2012), phosphorylation of proteins, action on ionic channels and transcription factors, production of thyroid hormones and crosslinking on extracellular matrix (Brieger et al., 2012).

The body tries to maintain redox homeostasis between the production of RONS and the capacity for their removal by antioxidant systems (Zuo et al., 2015), which allows the redox state to be re-established after temporary exposure to high concentrations of RONS and minimize the risk of a deteriorated redox homeostasis, which is an unbalanced state known as OS (Sies, 1986; Coyle and Puttfarcken, 1993; Liguori et al., 2018) (Figure 1). However, redox homeostasis is conditioned by the magnitude and duration of exposure to free radicals, since constant exposure can have a serious impact on intracellular signals or genetic expression, resulting in irreversible pathological consequences (Rhee et al., 2003), since most reactions of the body are dependent on the redox state (Tan et al., 2018).

Diseases associated with OS, such as neurodegenerative diseases, are related to aging (Liguori et al., 2018), a physiological stage accompanied by progressive loss of tissue and organ function (Flatt, 2012), changes in regulatory processes, decrease in the antioxidant capacity of the organism and irreversible tissue damage by RONS that compromises the achievement of a redox balance (Romano et al., 2010). The damage caused by oxidation depends on the defects of the enzymes involved in the redox signaling pathways (Tan et al., 2018).

Free radicals can cross the cells and cause modifications in the main macromolecules (lipids, proteins and nucleic acids), damaging their structure and altering their normal function (Salisbury and Bronas, 2015). Lipid peroxidation is associated with different disease states and is responsible for an unstable cell membrane, oxidation of low-density lipoproteins (LDL) and poly-unsaturated fatty acids (PUFAs). The oxidation of different amino acids such as lysine, arginine, proline and threonine results in protein dysfunction. Oxidative damage of DNA generates severe mutations and adverse effects on transcription, producing an RNA more vulnerable to oxidation. Chronic persistence of this situation is capable of causing cell death (Huiyong et al., 2011; Dizdaroglu and Jaruga, 2012).

OS plays an important role in chronic, inflammatory pathologies (Kehrer and Klotz, 2015), progressive brain damage and the pathogenesis of neurodegenerative diseases such as: Amyotrophic lateral sclerosis (ALS), Alzheimer’s disease (AD), Parkinson’s disease (PD), Multiple sclerosis (MS), Huntington’s disease (HD), and Spinocerebellar ataxia (SA) (Dias et al., 2013; Islam, 2017; Zewen et al., 2018) (Figure 1).

## Oxidative Stress and Neurodegenerative Diseases

The exact pathogenesis of neurodegenerative diseases remains unknown, although a complex and multifactorial origin has been established in which aspects such as genetic predisposition, certain endogenous factors and exposure to environmental factors are involved (Correia and Moreira, 2010; Correia et al., 2010; Coppedè and Migliore, 2015). ROS is a key factor in the etiology of these pathologies (Bhat et al., 2015) and in patients with a neurodegenerative disease, high levels of OS biomarkers have been observed (St-Pierre et al., 2006; Niedzielska et al., 2016). However, physiological concentrations of free radicals play an important role in normal brain function (De Silva and Miller, 2016; Scialò et al., 2017; Chen et al., 2018). Thus, ROS can contribute to: vascularization, cerebral perfusion, cellular signaling, synaptic plasticity, neurotransmitter secretion and cerebral vasodilation (Massaad and Klann, 2011; Grochowski et al., 2018; Neal and Richardson, 2018). A moderate increase of ROS secondary to mitochondrial activity produces preconditioning that leads to a neuroprotective function against harmful agents and is even preventive against massive ROS formation (Dirnagl and Meisel, 2008; Jou, 2008; Dirnagl et al., 2009). The origin of the problem lies in impaired redox homeostasis, which causes damage to cell membrane, compromising the viability and integrity of the central nervous system (CNS) (Van Horssen et al., 2011; Payne and Chinnery, 2015). The excess of ROS is related to changes in the microcirculation of the brain (Freeman and Keller, 2012; Staiculescu et al., 2014). O${}_{2}^{∙-}$ and H_2_O_2_ are able to cause a contraction of cerebral blood vessels (Allen and Bayraktunan, 2009). Increased levels of H_2_O_2_ are associated with increased pro-apoptotic agents in brain vascular cells (Li et al., 2003) and OS can alter cerebral vascular function through an interruption of NO^∙^ signaling and consequently, its vasodilatory and anti-inflammatory capacity (Miller et al., 2010; De Silva and Miller, 2016). In addition, ROS contribute to the maintenance of a proinflammatory state, secondary to secretion of cytokines and chemokines (Hsieh and Yang, 2013; Kehrer and Klotz, 2015).

The CNS is especially susceptible to oxidative damage (Cordeiro, 2014). The brain is largely formed by PUFAs with high sensitivity to lipid peroxidation (Nunomura et al., 2006), motor neurons are highly sensitive to OS (Simpkins and Dykens, 2008) and the CNS has a poor antioxidant capacity with low activity of protective enzymes such as glutathione peroxidase (GPx), catalase (CAT) or superoxide dismutase (SOD) and poor capacity for cell regeneration (Fischer and Maier, 2015; Kim et al., 2015; Formella et al., 2018). Although the brain only accounts for 2% of body weight (Mergenthaler et al., 2013) and mitochondrial density is higher in myocytes than in neuronal cells (Gandhi and Abramov, 2012), it is an organ with a high metabolic rate that can consume up to 20% of the total oxygen of the body (Moreira et al., 2009; Ronnett et al., 2009). This means it has 10 times higher energy and glucose consumption than the other tissues and a high dependence on mitochondrial energy production, responsible for the increase of ROS (Carvalho and Moreira, 2018). Taking into account the high rate of demand and energy consumption of the brain, the majority of mitochondrial mutations can affect the functioning of the CNS and have been associated with neurodegenerative diseases (Angelova and Abramov, 2018). Thus, mitochondrial dysfunction is currently seen as a “convergence point” in neurodegeneration (Correia et al., 2010; Bennett et al., 2014; Arun et al., 2016; Kurtishi et al., 2018).

Mitochondrial dysfunction compromises the energy supply of ATP to neurons, calcium homeostasis and leads to high levels of ROS that accelerate the mutation rate of mitochondrial DNA (mtDNA) and lipoperoxidation of neuronal membranes, causing decomposition of PUFAs and the formation of highly reactive end products: malondialdehyde (MDA) and 4-hydroxy-2-nonenal (HNE) (the most toxic species related to cell damage and apoptosis) (Petrozzi et al., 2007; Albarracín et al., 2012; Fritz and Petersen, 2013) (Figure 1). Accumulation of mutations in mtDNA causes an increase in oxidative damage, a decrease in energy rate and an increase in ROS. Thus, mitochondrial dysfunction generates a “vicious-cycle” responsible for neuronal damage, genetic mutations and metabolic stress, a situation that can lead to apoptosis (Van Houten et al., 2006; Selvaraji et al., 2019; Wang et al., 2019).

Mitochondrial dysfunction is considered as the main cause of neurodegenerative diseases pathogenesis (ALS, PD, AD, MS, HD) (Lin and Beal, 2006; Federico et al., 2012) (Figure 1). Postmortem studies in brains of patients with these diseases have found that mitochondrial dysfunction is a common event leading to the degeneration and death of neuronal cells (Schon and Manfredi, 2003; Bao and Swaab, 2018; Du et al., 2018).

Brain aging increases sensitivity to OS and decreases the effectiveness of antioxidant defenses, causing functional deficiencies, inflammation, decreased elasticity and greater susceptibility to the etiological factors involved in the pathogenesis of neurodegenerative disease (Federico et al., 2012; Guo et al., 2013). It also contributes to the accumulation of mutations in the mtDNA, malfunctions in the oxidative phosphorylation pathway and impaired redox homeostasis (Sarasija and Norman, 2018).

ROS have currently been proposed as one of the main contributors to the development and progression of neurodegenerative diseases (Morató et al., 2014). There is evidence that indicates that the increase in RONS concentration is a inducer of tissue damage, activation of microglia and astrocytes, neuronal dysfunction, neurodegeneration and cell death (Federico et al., 2012; Morató et al., 2014; Schieber and Chandel, 2014). Thus, a “vicious cycle” between OS, mitochondrial dysfunction, aging and neuroinflammation has been demonstrated (Witte et al., 2010; Van Giau et al., 2018). However, further studies are necessary to understand the physiopathological mechanisms implicated (Schetters et al., 2018) because it is unknown whether OS is a primary cause that induces the initiation of neurodegenerative diseases or if it is a side effect associated with the spread of damage to nerve cells (Jenner, 2003; Emerit et al., 2004).

## Amyotrophic Lateral Sclerosis

ALS, also known as Charcot’s disease or Lou Gehrig’s disease, was originally described by Jean-Martin Charcot and Joffrey (Chen et al., 2013; Niedzielska et al., 2016) and is the most common motor neuron disease (Rechtmann et al., 2015). It causes a selective loss of upper motor neurons (UMN) of the motor cortex and lower motor neurons (LMN) of the brainstem and spinal cord (Brown and Al-Chalabi, 2017; Valko and Ciesla, 2019).

Patients with ALS develop weakness, muscle denervation, atrophy and progressive paralysis of all muscles (bulbar and respiratory), dysphagia (Al-Chalabi and Hardiman, 2013; D’Amico et al., 2013; Gowland et al., 2019) and respiratory muscle weakness that leads to respiratory failure and death (Sferrazza-Papa et al., 2018). The survival rate is 3–5 years after the onset of symptoms (Andersen, 2006; Gordon, 2013; Coan and Mitchell, 2015; Wier et al., 2019).

The incidence and prevalence of ALS vary according to the geography and design of the study (Marin et al., 2017; Kaye et al., 2018) and there are differences between African–American and Hispanic populations (Gordon et al., 2013). The incidence is 2–3 cases for every 1.000.000 inhabitants/year and prevalence of 4.6 – 5 for every 100.000 inhabitants in Western European countries (Chiò et al., 2013; Talbot, 2016). A significant increase in the overall prevalence of ALS to 8.58 cases per 100.000 people is expected by the year 2020 and to 9.67 per 100,000 people by 2116. In the United Kingdom prevalence in the 2010 year was 1415 cases and is projected to increase to 1701 by 2020 and 2635 by 2116 (Gowland et al., 2019).

ALS typically occurs in white adults between the ages of 50–60 (Chiò et al., 2013; Mehta et al., 2018), and cases in children are very rare (Martin, 2010; Bäumer et al., 2014; Ingre et al., 2015). The age of onset is earlier in men than in women and men are more prone to spinal involvement, whereas bulbar involvement is more common in women (McCombe and Henderson, 2010).

ALS is classified into two types: familial ALS (fALS) and sporadic ALS (sALS). fALS represents approximately 5–10% of the cases and is related to heterogeneous mutations of a set of genes with an autosomal dominant inheritance pattern (Mitchell and Borasio, 2007). Up to 20% of cases of fALS is due to a mutation of the gene that encodes the enzyme Cu-Zn superoxide dismutase (SOD1). sALS occurs in up to 90–95% of the cases but its origin is still unknown (Martin, 2010; Andersen and Al-Chalabi, 2011; Pan et al., 2012). fALS and sALS can affect any voluntary muscle but their presentation, phenotype and progression are variable (Wei et al., 2019), hindering differential diagnosis (Swinnen and Robberecht, 2014; Ghasemi, 2016; Van Es et al., 2017). ALS may manifest double onset: involvement of the extremities (80% of cases) or bulbar involvement (20% of cases) (Parakh et al., 2013).

The death of motor neurons results in a characteristic clinical feature: spasticity, muscular weakness and atrophy, hyperreflexia, cramps, fasciculation, Babinski sign, loss of coordination, paralysis of voluntary musculature, dysphagia and difficulties in swallowing, speech and respiration (Weiduschat et al., 2014; Zufiría et al., 2016; Solleiro-Villavicencio and Rivas-Arancibia, 2018). It is associated with metabolic disorders such as weight loss, hypermetabolism and hyperlipidemia (Dupuis et al., 2011). fALS and sALS do not affect the senses (sight, smell, taste, hearing and touch) (Fiala et al., 2013).

Several factors have been identified related to the pathogenesis of ALS: OS, mitochondrial dysfunction, neuroinflammation, excitotoxicity due to an increase in the neurotransmitter glutamate, defect in axonal transmission and in the metabolism of RNA, apoptosis, cytoskeletal abnormalities, disruption of membrane trafficking, endoplasmic reticulum stress, protein misfolding and aggregation (Papadimitriou et al., 2010; Blokhuis et al., 2013; Peters et al., 2015; Bond et al., 2018; Yusuf et al., 2018) and cysteine modifications like oxidation or palmitoylation that contribute to a general aberration of cysteine residues proteostasis (Valle and Carrì, 2017). A relationship has been established between environmental conditions and the epidemiology of ALS: alcohol, tobacco, sedentary lifestyle, fungal and viral infections or exposure to electromagnetic radiation (Yu et al., 2014).

There are currently no effective treatments for ALS and therapy is limited to symptomatic and palliative treatment (Bhattacharya et al., 2019). Riluzole (Miller et al., 2012) and edaravone (Rothstein, 2017) are the only approved pharmacological therapies that increase the survival rate by 2–3 months (Dorst et al., 2017; Hodgkinson et al., 2018), but edaravone is only beneficial to a subset of people with early stage ALS (Abe et al., 2017). Other therapeutic routes indicate multidisciplinary treatment involving: nurses, dieticians, nutritionists, occupational therapists, physical therapists, psychologists, social workers and speech therapists (Gordon, 2013).

## Oxidative Stress and Amyotrophic Lateral Sclerosis

Presence of OS biomarkers and high levels of ROS have been determined in the CNS regions specifically in ALS patients (Golenia et al., 2014), which indicates that impaired redox homeostasis is a relevant factor and associated with the development and progression of neurodegeneration in fALS and sALS (Carrì et al., 2015; Petrov et al., 2017; Smith et al., 2017). High levels of certain types of ROS (H_2_O_2_ and O${}_{2}^{∙-}$) have been observed in affected cells (Beal et al., 2005).

Loss of oxidative control and excessive ROS production are particularly linked to the mutated forms of SOD1 (Ferri et al., 2006; Hooten et al., 2015; Angelova and Abramov, 2018), which can have up to 150 different types of mutations (Gamez et al., 2006; Al-Chalabi et al., 2013). SOD1 is an antioxidant enzyme that catalyzes the conversion of O${}_{2}^{∙-}$ into H_2_O_2_ and O_2_, and is key in the regulation of OS, cell damage (Mattiazzi et al., 2002; Li et al., 2011; Pehar et al., 2014), energy metabolism and cellular respiration (Saccon et al., 2013). Oxidation and/or misfolding of SOD1 results in a deterioration in the regulation of the above processes, mitochondrial dysfunction and increase in the production of superoxides (Israelson et al., 2010; Pickles et al., 2013, 2016; Richardson et al., 2013). Its mutation predisposes cellular organelles to oxidative damage (Deng et al., 2006; Ahtoniemi et al., 2008) and excessive levels of ROS that attack astrocytes (Islam, 2017). OS contributes to the aggregation of SOD1 (Brujin et al., 2004) enhancing mitochondrial dysfunction (Liu et al., 2004; Vijayvergiya et al., 2005).

The transgenic mouse model of ALS (SOD1^G93A^) has enabled the identification of pathogenic mechanisms and the establishment of a “vicious cycle” between: OS, mitochondrial dysfunction and deterioration of the ETC (Jung et al., 2002; Menzies et al., 2002; Pfohl et al., 2015; Xiao et al., 2018). Most of the oxidative species that are formed in the CNS are secondary to oxidative phosphorylation (Brookes et al., 2004; Balaban et al., 2005; Karam et al., 2017) and mitochondrial dysfunction is the largest source of ROS production, so is a clear sign of affectation of motor neurons in the spinal cord and the motor cortex (Bendotti et al., 2001; Kawahara et al., 2004; Loizzo et al., 2010; Cozzolino and Carrì, 2012). Mitochondrial damage causes an alteration in intracellular calcium levels and in normal functioning of the ETC (Bond et al., 2018) that contributes to the increase of ROS and to the activation of chain reactions that lead to greater oxidative damage, reinforcing the “vicious cycle” (Harraz et al., 2008; Federico et al., 2012; Rojas et al., 2015; Loeffler et al., 2016; Van Horssen et al., 2017) and increasing cellular susceptibility to apoptosis (Guegan et al., 2001; Iverson and Orrenius, 2004).

Changes in antioxidant defense markers have been observed in patients with ALS (Cova et al., 2010). However, the results are random, due to the wide heterogeneity, form of presentation and variability of the pathogenic mechanisms of the disease (Johnson et al., 2012). Low levels of reduced glutathione (GSH) in erythrocytes (Babu et al., 2008) and in the motor cortex (Ikawa et al., 2015) of patients with ALS, a systemic pro-inflammatory state (increased levels of IL-6 and IL-8) and an impaired antioxidant system (Ehrhart et al., 2015) have been highlighted. One study compared *in vivo* levels of GSH in the motor cortex of 11 ALS patients with those in 11 age-matched healthy volunteers and determined that GSH levels were 31% lower in ALS patients than in healthy volunteers (Weiduschat et al., 2014). Also, decreased glutathione levels caused motor neuron degeneration in the hSOD1^wt^ over-expressing mice model (hemizygous mice over-expressing wild-type hSOD1 at moderate levels) (Killoy et al., 2018) and accelerated motor neuron death in SOD1^G93A^ mice (Pehar et al., 2014). Catalase (CAT) activity is decreased in erythrocytes of sALS patients (Nikolic-Kokic et al., 2006; Babu et al., 2008) and is significantly decreased compared to neurologically healthy controls (Golenia et al., 2014).

Studies conducted *in vitro* indicated that oxidative damage to nerve cells (astrocytes and oligodendrocytes) was related to neurodegeneration (Pollari et al., 2014). Oxidative imbalance decreases the number of glial cells and the ability to transmit the axonal signal (Park et al., 2007).

Markers for OS have been determined in the cerebrospinal fluid (CSF), tissues, blood and urine of patients with ALS (Blasco et al., 2017). After postmortem analysis of neuronal tissues in sALS and fALS patients, an increase of OS biomarkers was noted in proteins, lipids and DNA (Beal, 2002; Agar and Durham, 2003; Kim et al., 2003; Turner et al., 2013; Bozzo et al., 2016). The most frequently studied biomarkers include: carbonylated and/or glycosylated proteins, lipid peroxidation and DNA damage (Shibata et al., 2002).

In the nervous tissues of ALS patients, high levels of carbonylated proteins have been measured in the motor cortex (Sasaki et al., 2000; Guareschi et al., 2012; Tan et al., 2014) and high levels of advanced products of protein oxidation in the plasma of the erythrocytes of sALS patients (LoGerfo et al., 2014). In ALS-mouse models, non-functional proteins secondary to the oxidation of amino acid residues by peroxynitrite have been observed (Yoshino and Kimura, 2006). In addition, oxidative damage results in the aggregation of TDP-43, the major disease-associated protein involved in the pathogenesis of ALS (Moujalled et al., 2017; Oberstad et al., 2018) that promotes OS in neuronal cells (Duan et al., 2010; Cohen et al., 2012, 2015; Magrané et al., 2014) (Figure 1).

CNS lipids are susceptible to oxidative damage (Niki, 2014) and lipoperoxidation generates high levels of HNE, MDA (Radak et al., 2011) and F2-isoprostane (F2-IsoPS) (Milne et al., 2007; Milatovic et al., 2011; Van’t Erve et al., 2017) (Figure 1). In the CSF of patients with ALS, high levels of HNE and 3-nitrotyrosine (3-NT) have been obtained (Simpson et al., 2004; Duffy et al., 2011; Perluigi et al., 2012; Ayala et al., 2014; Santa-Cruz and Tapia, 2014).

The guanine contained in DNA is highly susceptible to oxidation and acts as a target for ROS (Lee et al., 2007; Chang et al., 2008). 8-oxo-deoxyguanosine (8-OHdG) can be considered as a specific biomarker of oxidation of the motor cortex in ALS (Cooke et al., 2000, 2002; Ihara et al., 2005; Mitsumoto et al., 2008) (Figure 1). Increased levels of 8-OHdG and isoprostanoids have been observed in the urine of subjects affected by the disease compared to the control group (Miller et al., 2014).

The evaluation of GPx activity, glutathione reductase (GR), SOD, total serum antioxidant status (TAS), MDA and 8-OHdG in ALS patients, found a significant decrease in TAS levels and an increase of 8-OHdG and MDA levels, together with significantly higher oxidized/reduced glutathione (GSSG/GSH) ratio and IL-6 and IL-8 (Blasco et al., 2017).

## Antioxidants and Amyotrophic Lateral Sclerosis

The evidence implicates free radicals and OS as factors related to the pathogenesis of ALS. Interest has focused on substances with antioxidant potential such as: vitamin E, carotenes, flavonoids, resveratrol, epigallocatechin gallate, curcumin, Co-enzyme Q10, melatonin and edaravone as useful agents in the management of this disease (Table 1).

**TABLE 1:** Antioxidant compounds used in the treatment of ALS.

Antioxidant	Features	Molecular mechanisms	Curative effects and treatment object	References
Vitamin E	- Lipophilic antioxidant - Ability to cross cell membrane	- Protection against lipoperoxidation - Protection against ROS and RNS	- Delay in the clinical onset of the disease - Increase GSH levels in plasma - Lower risk of dying by ALS - Significant decrease in the risk of the disease - Lower ALS rates	Gurney et al., 1996; Desnuelle et al., 2001; Ascherio et al., 2005; Veldink et al., 2007; Wang H. et al., 2011
Carotenes	- Natural pigments - Different types: ß-carotene, lutein, astaxanthin and lycopene	Antioxidant and neutralizing properties against ROS	- Help prevention and/or delay the onset of ALS - Reduced risk of ALS - Treating neuroinflammation and apoptosis	Fitzgerald et al., 2013; Krishnaraj et al., 2016
Flavonoids	- Natural substances of fruits and vegetables - Different types: 7,8-DHF, fisetin and quercetein	- Protection against ROS - Modulate metabolic pathways	- Improve motor deficits and enhance lower neuronal survival - Reduction of intracellular ROS levels - Reduction of motor neuron loss - Improve motor activity and survival rate - Inhibition of aggregation and misfolding of SOD1	Korkmaz et al., 2014; Ip et al., 2017; Wang et al., 2018
Resveratrol	- Natural polyphenolic compound - In grapes, peanuts and berries - Produced in plants in response to mechanical injury, fungal infection, and UV radiation - Scavenger of free radicals	- Interacts with mutant SOD1 (G93A) protein - Up-regulates SIRT1 - Down-regulation of AMPK/SIRT1 signaling in bone marrow mesenchymal stem cells	- Delays the onset of ALS - Increases survival of spinal motor neurons - Preserves the function of the lower and upper motor neuron - Attenuates the loss of motor neurons - Improves muscle atrophy - Improves mitochondrial function of muscle fibers	Mancuso et al., 2014a, b; Song et al., 2014
Epigallocatechin gallate	- Catechin present in green tea - Crosses the blood-brain barrier	- Modulates mitochondrial responses to OS - Protection against lipoperoxidation - Changes intracellular signals - Reduces the concentration of NF-kB caspase-3 and iNOS	- Prevents OS-induced death - Delays the outbreak or progression of ALS - Delays the onset of the disease - Prolongs useful life - Increases the number of motor neurons - Decreases the activation of microglia	Koh et al., 2004, 2006; Xu et al., 2006
Curcumin	- Natural and liposoluble dye - Obtained from turmeric - Chemical instability - Low oral bioavailability - Low water solubility rate - Different types: DMC	- Activates Nrf2 - Decreases intracelullar ROS levels - Eliminates excitability induced by TDP-43 - DMC decreases mitochondrial dysfunction	- Improves survival - Decrease in ALS progression and reduction of oxidative damage	Ahmadi et al., 2018; Chico et al., 2018
Co-enzyme Q10	- Endogenous antioxidant	- Cofactor of the ETC - Action in redox balance - Improves mitochondrial dysfunction	- Increases survival rate	Matthews et al., 1998; Beal, 2002
Melatonin	- Amphiphilic molecule - Potent antioxidant	- Antioxidant - Regulator of mitochondrial bioenergetic function	- Delays the progression of the disease - Increases the survival rate	Zhang et al., 2013 Weishaupt et al., 2006
Edaravone	- Low-molecular-weight antioxidant drug - Intravenously administered - Free radical scavenger - Safe - Crosses the blood-brain barrier easily - High brain penetration capacity - Amphiphilic capacity - Scavenges lipid and water soluble peroxyl radicals and chain- carrying lipid peroxyl radicals	- Enhances prostacycling production - Traps hydroxyl radical and quenches active oxygen	- Deletes lipid peroxides and hydroxyl radicals during cerebral ischemia - Protects nerve cells within or around the ischemic region from free radical damage - Ameliorates OS and suppresses degeneration of spinal motor neurons - Anti-inflammatory and protective effects on neurons, microglia, astrocytes and oligodendrocytes - Delays the progression of functional motor disturbances	Ikeda and Iwasaki, 2015; Banno et al., 2005; Yoshino and Kimura, 2006; Miyamoto et al., 2013; Abe et al., 2014; Bailly, 2019

### Vitamin E

Vitamin E (α-tocopherol) is one of the most studied lipophilic antioxidants due to its ability to cross the cell membrane and the protection that it provides against lipoperoxidation (Butterfield et al., 2002; Di Matteo and Esposito, 2003), ROS and RNS (Barber and Shaw, 2010) (Table 1). The studies on nutritional supplementation with this vitamin have obtained contradictory results.

This antioxidant has been associated with a delay in the clinical onset of the disease (Table 1) in transgenic mice that express mutant copies of the gene encoding SOD1 (an animal model of ALS) (Gurney et al., 1996). Measurements of the erythrocyte activity of the three main radical scavenging enzymes (SOD1, CAT, and GPx) indicated that vitamin E supplementation (300–3000 U/day) in 14 ALS patients did not affect the activity of the three enzymes (Przedborski et al., 1996). One study showed an accumulation of vitamin E in the spinal cord and increased MDA levels over the lifetime of the mouse compared to non-transgenic mice (Hall et al., 1998).

In a double-blind placebo-controlled randomized clinical trial, vitamin E did not appear to affect survival and motor function in ALS. However, after 3 months of treatment with vitamin E and riluzole, an increase in plasma GSH levels (Table 1) and a decrease in plasma thiobarbituric acid reactive species (TBARS) levels were observed, markers typically altered in the plasma of the patients with ALS (Desnuelle et al., 2001). Vitamin E was associated with a slower progression of ALS (Orrell et al., 2004) and in one study it was observed that regular use of vitamin E supplements was associated with a lower risk of dying by ALS (Ascherio et al., 2005) (Table 1).

Other similar studies did not detect significant differences between placebo and the group treated with α-tocopherol and indicated that there are insufficient results to claim that megadoses with vitamin E are effective in slowing the progression of the disease (Graf et al., 2005). Supplementation with 600 mg/day of vitamin E did not show differences with respect to the placebo group (Galbussera et al., 2006). In a case-control study with 132 patients suffering from ALS, a significant decrease in the risk of the disease (Table 1) was observed when the intake of vitamin E was higher than the average (Veldink et al., 2007). Supplementation with vitamin E over a prolonged period was associated with lower ALS rates (Wang H. et al., 2011) and a study that included 50 cases of male ALS patients concluded that in men with baseline serum α-tocopherol below the average, there was a non-significant decrease in ALS risk in those receiving α-tocopherol supplementation (50 mg/day) compared to those not receiving α-tocopherol. If baseline serum α-tocopherol was above the average, α-tocopherol supplementation had no effect on the risk of ALS. In this case, α-tocopherol supplementation did not have a significant protective effect on ALS risk (Michal-Freedman et al., 2013).

### Carotenes

Carotenes are natural pigments, responsible for the orange, red (Krinsky and Johnson, 2005), yellow or green color of fruits and vegetables (Guest and Grant, 2016) with antioxidant and neutralizing properties against ROS (Fiedor et al., 2012; Nisar et al., 2015) (Table 1).

There is a beneficial association between ALS and the intake of carotenes (Okamoto et al., 2009; Nieves et al., 2016). Thus, their consumption could help the prevention and/or delay the onset of ALS (Fitzgerald et al., 2013) (Table 1) but in a case-controlled study with 77 Koreans diagnosed with ALS, it was determined that dietary intake of carotenes was negatively associated with ALS (Jin et al., 2014). A study conducted in 5 cohorts determined that a higher intake of these pigments was associated with a reduced risk of ALS and that high dietary intakes of ß-carotene and lutein were inversely associated with the risk of suffering from this disease. Astaxanthin and lycopene have shown a beneficial effect in ALS (Longecker et al., 2000; Isonaka et al., 2011) but in a study with the SOD1^G93A^ mice model of ALS, a tomato-enriched food (rich in lycopene) did not affect disease onset and survival (Esposito et al., 2007). β-carotene could serve as a potential therapeutic molecule for treating neuroinflammation and apoptosis in ALS patients (Krishnaraj et al., 2016) (Table 1).

### Flavonoids

Flavonoids are natural substances mainly present in fruits and vegetables (He et al., 2017) that have a protective effect against ROS (Cao et al., 1997), modulate the activity of different metabolic pathways (Mansuri et al., 2014) (Table 1) related to the reduction of cognitive damage and neuronal dysfunction (Vauzour et al., 2008) and suppress neuroinflammation (Solanki et al., 2015).

7,8-dihydroxyflavone (7,8-DHF) has neuroprotective and regulatory properties on neuromuscular transmission (Mantilla and Ermilov, 2012). Chronic administration of 7,8-DHF significantly improved motor deficits and enhanced lower neuronal survival in the transgenic ALS mouse model (Korkmaz et al., 2014) (Table 1).

Treatment with fisetin reduced intracellular ROS levels, motor neuron loss and improved motor activity and survival rate in three different hSOD1-related mutant models of ALS (Drosophila expressing hSOD1^*G*85R^, hSOD1^G93A^NSC34 cells and transgenic mice hSOD1^G93A^) (Wang et al., 2018) (Table 1).

Quercetin is an abundant flavonoid in the diet (onion, apple, broccoli and berries) (Esposito et al., 2002) that has been shown to reduce mitochondrial damage in various animal models of OS. Treatment with quercetin could be a therapeutic strategy for attenuating neuronal death against aluminum-induced neurodegeneration in the rat hippocampus (Sharma et al., 2016). Quercetin and its derivative, quercetin 3-β-D-glucoside (Q3BDG), could be therapeutic inhibitors of the aggregation and misfolding of SOD1 associated with ALS (Ip et al., 2017) (Table 1).

### Resveratrol

Resveratrol (RES) can interact with mutant SOD1 (G93A) protein (a distinctive feature of ALS) (Julien, 2007; Srinivasan and Rajasekaran, 2018; Laudati et al., 2019) and has positive effects by up-regulating sirtuin 1 (SIRT1) expression in the mutant hSOD1-G93A-bearing motor neuron-like cell culture model of ALS (Wang J. et al., 2011) (Table 1). It delays the onset of ALS, increases the survival of spinal motor neurons, preserves the function of the lower and upper motor neuron (Mancuso et al., 2014a, b), attenuates the loss of motor neurons and improves muscle atrophy and mitochondrial function of muscle fibers in a SOD1^G93A^ mouse model of ALS (Song et al., 2014) (Table 1). One study showed that bone marrow mesenchymal stem cells from ALS patients showed down-regulation of AMPK/SIRT1 signaling, which was recovered by treatment with RES (Yun et al., 2019) (Table 1).

### Epigallocatechin Gallate

Epigallocatechin gallate (EGCG) is a catechin present in green tea (Chung et al., 2010) and to which an antineurodegenerative and antioxidant effect is attributed (Nie et al., 2002; Panickar et al., 2009), especially on the motor neurons (Koh et al., 2006) because it crosses the blood-brain barrier and modulates mitochondrial responses to OS (Bedlack et al., 2015) (Table 1). It also protects against lipoperoxidation (Table 1) in *in vitro* studies by exposing ROS to the phospholipids of the cell membrane bilayer (Terao et al., 1994). It prevents OS-induced death of mutant SOD1 motor neuron cells by alteration of cell survival and death signals (Koh et al., 2004). Treatment with EGCG could delay the outbreak or progression of ALS through changes in intracellular signals, increases survival signals (like PI3-K and Akt) and reduces death signals (like GKS-3ß, cytosolic cytochrome c, activated caspase-3 and cleaved poly ADP-ribose polymerase) (Levites et al., 2002; Mandel et al., 2003, 2005; Koh et al., 2006) (Table 1). Oral administration of 10 mg/kg of EGCG from a presymptomatic stage delays the onset of the disease and prolongs useful life, in addition to increasing the number of motor neurons, decreasing the activation of the microglia, reducing the concentration of NF-kB, caspase-3 and iNOS in a transgenic mouse model of ALS (Xu et al., 2006) (Table 1).

### Curcumin

Curcumin is a natural and liposoluble dye obtained from turmeric (Table 1). It has neuroprotective effects and provides protection against OS (Kim et al., 2012), mitochondrial dysfunction, inflammation and protein aggregation (Ullah et al., 2017; Rakotoarisoa and Angelova, 2018; Ghasemi et al., 2019).

Curcumin activates nuclear factor erythroid 2-related factor (Nrf2) target genes in primary spinal cord astrocytes, decreases the level of intracellular ROS and attenuates oxidative damage and mitochondrial dysfunction (Jiang et al., 2011) (Table 1). It eliminates the excitability induced by TDP-43 (the major pathological protein in sporadic ALS) in a motor neuron-like cellular model of ALS (Mackenzie and Rademakers, 2008; Dong et al., 2014). Dimethoxy curcumin (DMC) could decrease mitochondrial dysfunction in mutated TDP-43 stably transfected cell lines (Lu et al., 2012) (Table 1). It binds to the pre-fibrillar aggregates of SOD1, altering its amyloidogenic pathway and decreasing cytotoxicity (Bhatia et al., 2015). Curcumin may improve survival in patients with ALS, especially those with bulbar involvement (Ahmadi et al., 2018). In a double-blind therapeutic trial, treatment with curcumin showed a decrease in ALS progression and a reduction of oxidative damage (Chico et al., 2018) (Table 1).

A study indicates that a drug delivery system based on curcumin will be proposed for the treatment of ALS (Tripodo et al., 2015) but others indicate that the use of curcumin as therapy in ALS has disadvantages: chemical instability and low oral bioavailability and water solubility rate (Rakotoarisoa and Angelova, 2018) (Table 1). Therefore, it is necessary to develop new technologies to overcome these limitations (Liu et al., 2016).

### Co-enzyme Q10

Co-enzyme Q10 (CoQ10) is an endogenous antioxidant and a cofactor in the ETC (Petrov et al., 2017) involved in redox balance (Yamamoto, 2016) (Table 1). In the SOD1 transgenic mouse model of ALS, supplementation with CoQ10 extended survival by 6 days and increased brain mitochondrial concentration compared to controls (Matthews et al., 1998; Strong and Pattee, 2000; Beal, 2002) (Table 1). After comparing the plasma redox status of CoQ10 in 20 sALS patients with those in healthy age/sex-matched controls, a significant increase in the oxidized form of CoQ10 in sALS was observed (Sohmiya et al., 2005). A study conducted on 31 subjects, showed that treatment with megadoses of 3000 mg/day of CoQ10 over 8 months could improve mitochondrial dysfunction in ALS (Ferrante et al., 2005). However, one study suggested that serum CoQ10 concentrations are unrelated to the risk of ALS (Molina et al., 2000; Kaufmann et al., 2009).

### Melatonin

Melatonin is an amphiphilic molecule that has been identified as a potent antioxidant therapeutic agent in neurodegenerative diseases (Polimeni et al., 2014) associated with mitochondrial dysfunction (Ganie et al., 2016) (Table 1). A study indicated that melatonin could be a candidate for neuroprotection in ALS (Jacob et al., 2002). In the SOD1^G93A^ transgenic mouse model of ALS, high doses of melatonin administered orally delayed the progression of the disease and increased the survival rate (Weishaupt et al., 2006; Zhang et al., 2013) (Table 1). However, evidence suggests that intraperitoneal melatonin is not neuroprotective and may exacerbate neurodegeneration (Dardiotis et al., 2013). Clinical trials employing melatonin in the range of 50–100 mg/day are required before its relative merits as a neuroprotective agent are definitively established (Pandi-Perumal et al., 2013). Recent studies suggest that riluzole but not melatonin ameliorates acute motor neuron degeneration and moderately inhibits SOD1-mediated excitotoxicity induced disrupted mitochondrial calcium signaling in ALS (Jaiswal, 2017).

### Edaravone

Edaravone is a low-molecular-weight antioxidant drug (Radicava^®^), administered intravenously (Petrov et al., 2017) which acts as a free radical scavenger (Jackson et al., 2019) (Table 1). In 2015, edaravone was approved for ALS treatment in Japan (Okada et al., 2018) and by the Food and Drug Administration of United States in 2017 (Watanabe et al., 2018).

Edaravone easily crosses the blood-brain-barrier and displays a high brain penetration capacity (Jin et al., 2017). Its amphiphilic capacity allows edaravone to scavenge both lipid and water soluble peroxyl radicals and chain-carrying lipid peroxyl radicals (Nagase et al., 2016) (Table 1).

The antioxidant mechanisms of edaravone are: enhancement of prostacyclin production, hydroxyl radical trapping and quenching of active oxygen (Sawada, 2017) (Table 1). Edaravone eliminates lipid peroxides and hydroxyl radicals during cerebral ischemia, protects nerve cells within or around the ischemic region from free radical damage (Abe et al., 2014), ameliorate OS and suppress degeneration of spinal motor neurons (Ikeda and Iwasaki, 2015). It is attributed anti-inflammatory (Bailly, 2019) and protective effects in neurons, microglia, astrocytes and oligodendrocytes (Banno et al., 2005; Miyamoto et al., 2013) (Table 1).

Investigation of the safety and efficacy of edaravone in 20 ALS patients who received this antioxidant intravenously indicated that this drug is safe and may delay the progression of functional motor disturbances by reducing OS (Yoshino and Kimura, 2006) (Table 1).

## Nicotinamide Riboside and Neurodegenerative Diseases

### NAD^+^ Role and Levels

**FIGURE 2:**
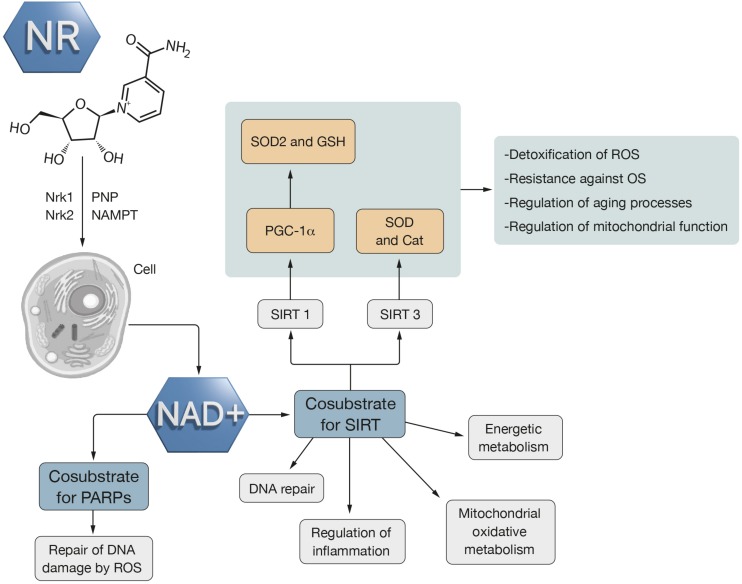
Nicotinamide riboside action mechanism. NR gets into the cell and there, it is converted into NAD^+^ through two mechanisms. One of them is Nrk1 and Nrk2 and the other is PNP and NAMPT. NAD^+^ is a cosubstrate of PARPS which is related to repair of DNA damage by ROS. NAD^+^ is also SIRT cosubstrate which is associated with energy metabolism, inflammation regulation, DNA repair and mitochondrial metabolism. The activation of SIRT increases the resistance against OS through an increase in metabolic pathways that detoxify ROS, like SOD2 and Cat. SIRT1 activates PGC-1α which involves an increase of antioxidant defense through SOD2 and GSH. SIRT3 activates SOD2 and Cat. Thus, SIRT regulates mitochondrial function and aging processes as well as is involved in ROS detoxification.

NAD^+^ is a coenzyme that takes part in critical redox reactions (its reduced form is NADH) for the operation of mitochondrial metabolism (Berger et al., 2004; Yoshino et al., 2018). Is an important biological mediator due to its many essential functions for survival: redox reactions, signaling pathways, energy metabolism, mitochondrial function, calcium homeostasis, DNA repair, gene expression (Guarente, 2014; Yaku et al., 2018), brain metabolism, neurotransmission, learning, memory, axonal neuroprotection (Araki et al., 2004; Conforti et al., 2006; Gong et al., 2013) and participation in the NAD^+^/PARP/SIRT1 axis related to aging (Mendelsohn and Larrick, 2017) (Figure 2).

NAD^+^ can act as a cosubstrate for sirtuins (SIRT) (Verdin, 2015), class III histone deacetylase enzymes whose activity is dependent on NAD^+^ levels (Tang, 2016; Jęśko et al., 2017) and for poly(ADP-ribose) polymerases (PARPs), a family of proteins essential for repairing DNA damaged by ROS (Shen et al., 2015) (Figure 2).

Sirtuins are related to the metabolic status of cells and are key in cellular metabolism, regulation of the expression of certain genes (O’Callaghan and Vassilopoulos, 2017), energy and mitochondrial metabolism (Covington and Bajpeyi, 2016), inflammation and DNA repair (Tang, 2017). They can be found in the cytoplasm, nucleus and mitochondria (Kupis et al., 2016). In neurodegenerative diseases and in aging they organize the response to OS and damage to DNA and are related to the functionality of the respiratory machinery and the production of ROS in different tissues (Huang et al., 2010).

Activation of SIRT increases resistance to OS (Haigis and Sinclair, 2010) through an increase in metabolic pathways responsible for the detoxification of ROS (Salvatori et al., 2017) such as superoxide dismutase 2 (SOD2), isocitrate dehydrogenase 2 (IDH2) and CAT (Tennen and Chua, 2011). They improve metabolic capacity, promote mitochondrial oxidative metabolism and facilitate repair of DNA damage (Haigis and Sinclair, 2010) (Figure 2).

SIRT1 acts on the pathway of the peroxisome proliferator-activated receptor gamma coactivator 1-alpha (PGC-1α) (Alquilano et al., 2013; Tang, 2016), a fundamental regulator of energy metabolism (Bayer et al., 2017; Thirupathi and de Souza, 2017) and mitochondrial dynamics (Singh et al., 2016) associated with an increase in antioxidant defenses through SOD2 and GSH (Liang and Ward, 2006; Zhong and Xu, 2008; Wang et al., 2015) (Figure 2). Reduction of SIRT1 activity compromises oxidative metabolism and antioxidant capacity, affecting complex I of the ETC, mitochondrial function and biogenesis (Rodgers et al., 2005). This effect has been observed in aging and different pathologies: neurodegenerative diseases, metabolic disorders and cancer (Rass et al., 2007; Hou et al., 2017b; Jęśko et al., 2017). In ALS, alterations in SIRT1 levels have been determined in postmortem tissues from patients (Körner et al., 2013) and in mouse models of ALS (Han et al., 2012).

Sirtuin 3 (SIRT3) regulates mitochondrial function and the aging processes (Buck et al., 2017) by activating SOD2 and CAT (Salvatori et al., 2017) that are involved in the detoxification of ROS (Qiu et al., 2010; Kida and Goligorsky, 2016) (Figure 2).

The decrease in NAD^+^ levels may be secondary to a defect in its biosynthesis or to depletion (Imai and Guarente, 2014) and leads to a deficiency in the activities of SIRTs in the nucleus and mitochondria (Chang and Guarente, 2013; Gomes et al., 2013; Mendelsohn and Larrick, 2014; Frederick et al., 2016), reduces the mitochondrial unfolded protein response (Mendelsohn and Larrick, 2017), disrupts ATP biosynthesis, decreases the ability to pump calcium against the intracellular gradient (Camandola and Mattson, 2011), disrupts calcium homeostasis, increases excitotoxicity (Rzheshevsky, 2014) and has deleterious effects on muscle health (Goody and Henry, 2018).

The lower bioavailability of NAD^+^ levels is involved in many diseases such as neurodegenerative pathologies (Essa et al., 2013; Johnson and Imai, 2018) and affects the production of energy, lowers the levels of ATP and limits the protection of SIRT1 (Houtkooper et al., 2010; Cantó et al., 2012; Imai and Guarente, 2014), an aspect that could worsen in these diseases because of its progressive nature (Cantó et al., 2015). NAD^+^ levels have been shown to decrease with age, leading to low levels in the brain (Zhu et al., 2015). Several studies have demonstrated that NAD^+^ metabolism is involved in neuronal function and in the pathophysiology of neurodegenerative diseases (Hikosaka et al., 2019) and that NAD^+^ levels in tissues can produce beneficial therapeutical effects in this type of diseases (Aman et al., 2018; Kulikova et al., 2018).

The activation of metabolic pathways related to mitochondria and the production of energy through the NAD^+^ and SIRT1 is currently suggested as a therapeutic strategy (Yang and Sauve, 2016; Tang, 2017; Rajman et al., 2018; Zhang and Sauve, 2018).

Recovery and/or increase of NAD^+^ levels can protect skeletal muscle from progressive deterioration (Fletcher et al., 2017), reverse the damage to cerebral energy metabolism, increase the protection against OS (López-Otín et al., 2013; Brown et al., 2014; Verdin, 2015), promote the activity of SIRTs and proteins involved in mitochondrial function (Bonkowski and Sinclair, 2016), confers resistance against peroxide toxicity, decreases mitochondrial ROS (Harlan et al., 2016), protection against neurodegeneration (Sasaki et al., 2006) and upkeep of dependent enzymes that are involved in synaptic plasticity and neuronal stress resistance (Lautrup et al., 2019).

Therefore, the repletion of NAD^+^ levels using precursors can ameliorate this age-associated functional defects (Imai and Guarente, 2014), helping to reverse the pathogenic processes characteristic of neurodegenerative diseases (Zhang et al., 2016).

### Precursors of NAD^+^

The precursors of NAD^+^ include nicotinamide mononucleotide (NMN), nicotinamide (NAM), nicotinic acid (NA) and nicotinamide riboside (NR) (Harlan et al., 2016). Most of the NAD^+^ precursors are more stable and have higher ability to enter neurons than NAD^+^ (Sasaki et al., 2006). NMN and NR are the most used and both have shown an increase in NAD^+^ levels in the cell (Nikiforov et al., 2011) (Table 2). Most evidence concludes that NR has a greater ability to stimulate a significant increase in NAD^+^ levels (Cantó et al., 2012) and advantages over the use of other precursors (Table 2).

**TABLE 2:** Comparison between the different NAD^+^ precursors.

NAD^+^ precursor	Advantages	Disadvantages	References
Niacin (NA)	- Prevents Pellagra - Regulation of lipid levels (total cholesterol, triglycerides and LDL)	- Produces cutaneous flushing - Not a NAD^+^ precursor in the majority of the cells	Davidson, 2008; Guille et al., 2008; Prousky et al., 2011
Nicotinamide mononucleotide (NMN)	- One of the most used - Has shown an increase in NAD^+^ levels in the cell	- No consensus on how is transported to the cell	Nikiforov et al., 2011; Grozio et al., 2019; Schmidt and Brenner, 2019
Nicotinamide (NAM)	Can be a stimulator in cells	Smaller increase in NAD^+^ compared to NR	Cantó et al., 2012
Nicotinamide riboside (NR)	- Action on mammalian cells - Minimal toxicity - High bioavailability - High capacity to cross the blood- brain barrier - Supports neuronal NAD^+^ synthesis - Greater ability to stimulate a significant increase in NAD^+^ levels and intermediate precursors	No evidence	Yang H. et al., 2007; Bogan and Brenner, 2008; Cantó et al., 2012; Trammell et al., 2016; Airhart et al., 2017; Martens et al., 2018

NA prevents Pellagra (Prousky et al., 2011) and has positive effects on the regulation of lipids (total cholesterol, triglycerides and LDL) (Guille et al., 2008) (Tabla 2). However, produces cutaneous flushing and does not work as NAD^+^ precursor in the majority of cells (Davidson, 2008) (Table 2). One of the most important advantages of NR over NA is that supports neuronal NAD^+^ synthesis (Bogan and Brenner, 2008) and has action on several types of cultured mammalian cells, including mouse and human cells (Yang H. et al., 2007) (Table 2).

There is no consensus on how NMN is transported to the cell (Grozio et al., 2019; Schmidt and Brenner, 2019) (Table 2). However, NR is five times more effective than NMN in increasing intracellular NAD^+^ levels in skeletal muscles (Oakey et al., 2018) (Table 2).

After comparing NR with NA and NAM in mice, NR showed a significant higher peak of NAD^+^ concentration and a significant increase of intermediate NAD^+^ precursors including NMN, NA mononucleotide and NA adenine dinucleotide (Trammell et al., 2016) (Table 2). NR has a different time course compared to NAM and NA and produces more NAD^+^ than the other precursors in equivalent doses (Trammell et al., 2016). NR increases the ratio of NAD^+^/NADH more significantly compared NMN, NAM and NA and this can contribute to improving the oxidative capacity of mitochondria (Cantó et al., 2012) and to decrease the rate of oxidative damage against DNA and mitochondrial stress (Ramamoorthy et al., 2012; Srivastava, 2016; Dolopikou et al., 2019) (Table 2).

NR is a nucleoside that incorporates a nicotinamide and a ribose (Belenky et al., 2007; Chi and Sauve, 2013) group. It is a trace nutrient known as vitamin B3 (Lanska, 2012), available in certain foods (dairy products, fish, eggs, and vegetables) (Minto et al., 2017), nutritional supplements and fortified foods (Colbourne et al., 2013). Its effects are associated with energy metabolism and neuroprotection (Chi and Sauve, 2013). When the NR enters the cells it is converted to NAD^+^ by at least two types of metabolic pathway. The first requires the participation of the nicotinamide riboside kinases (NRKs) (Chi and Sauve, 2013) in two of its isoforms (NRK1 and NRK2) (Bieganowski and Brenner, 2004) and the second requires the action of purine nucleoside phosphorylase (PNP) and nicotinamide phosphoribosyltransferase (NAMPT) (Burgos et al., 2013) (Figure 2).

The conversion of NR into NAD^+^ has been observed in animal tissues of muscle and brain (Chi and Sauve, 2013) and treatment by exogenous supplementation of NR is able to increase intracellular concentrations of NAD^+^ and promote its beneficial effects (Braidy et al., 2008) (Figure 2). NR enhances protection against aging and related diseases, with positive results on longevity in multiple animal models (Mouchiroud et al., 2013; Poljsak and Milisav, 2016) and has been recently reported that NR protects against excitotoxicity induced axonal degeneration (Vaur et al., 2017). The increase of NAD^+^ levels by supplementation with precursors such as NR improves mitochondrial and muscular function and the function of neuronal cells in mice (Mendelsohn and Larrick, 2017). A study performed in mice supplemented nutritionally with 400 mg/kg/day of synthetic NR showed an increase in NAD^+^ levels in muscle and liver tissue (Cantó et al., 2012). As NR increases the NAD^+^/NADH ratio, it could be related to an improvement of the oxidative capacity of the mitochondrial pathway and could be a therapeutic strategy of interest in those diseases that are associated with mitochondrial dysfunction and OS such as neurodegenerative diseases (Cantó et al., 2012). Improvement of NAD^+^ levels provides greater mitochondrial resistance against OS (Yang T. et al., 2007) and prevents cell death due to the protective function of SIRT3 (Hafner et al., 2010), which stimulates SOD, responsible for enhancing the “detoxification” of ROS (Chen et al., 2011) (Figure 2).

Supplementation with NR is safe in mice and humans and has minimal toxicity, high bioavailability and high capacity to cross the blood-brain barrier (Trammell et al., 2016; Airhart et al., 2017; Martens et al., 2018) (Table 2).

### Nicotinamide Riboside

Neurodegenerative diseases are associated with a progressive decrease in muscle mass and strength and an increase in their weakness (González et al., 2017; Van Damme et al., 2017; Dahlqvist et al., 2019). Muscle contractility dysfunction precedes the loss of motor unit connectivity in the SOD1^G93A^ mouse model of ALS (Wier et al., 2019) with neurodegeneration being one of the determinant risk factors for muscle quality (Di Pietro et al., 2017; Moon et al., 2018). Treatment with NR improves muscle function in aged mice (Cantó et al., 2012) and has benefits on muscle strength and skeletal muscle resistance in knock out mice that have specific elimination of NAMPT and a decrease of 85% in the intramuscular content of NAD^+^ (Frederick et al., 2016). This would indicate that the maintenance of NAD^+^ homeostasis is critical for muscle mass and its contractile function (Mendelsohn and Larrick, 2014; Frederick et al., 2016). NR induces rejuvenation in muscle and brain stem cells (Ryu et al., 2016) and an increase in neurogenesis and muscle function (Zhang et al., 2016). NAD^+^ is a fundamental modulator of muscle senescence and after treatment with NR, there is an acceleration of muscle regeneration in both young and old mice and an improvement in running times, maximum distances, grip strength of the extremities of aged mice and increased expression of cell cycle genes (Zhang et al., 2016).

Levels of PGC-1α are decreased in Alzheimer’s disease (AD) and this is related to the formation of ß-amyloid plaques and greater deposition of this substance (main characteristics of AD) (Wu et al., 2006; Quin et al., 2009). Studies conducted in animal models of AD established a relationship between the levels of NAD^+^ and a decrease in the toxicity of the amyloid substance (Kim et al., 2007; Quin et al., 2009). For this reason, the therapeutic strategy based on nutritional supplementation with NR could be of interest in AD cases to stop the onset and progression of the dementia (Rodgers et al., 2005) and to improve cognitive function and synaptic plasticity by promoting the function of PGC-1α as demonstrated in Tg2576 mice (mouse model of ALS) (Gong et al., 2013). After analyzing the effect of NR on the physiopathology and cellular mechanisms in the 3xTgAD/Polß^±^ mouse model (mice deficient in DNA repair that exacerbates the main characteristics of AD in humans) it was observed that supplementation with NR significantly increased the NAD^+^/NADH ratio, reduced neuroinflammation, apoptosis and damage to DNA, increased neurogenesis, restored synaptic plasticity, improved learning capacity and reversed memory deficits (Hou et al., 2017a). Treatment with NR improves cognition in transgenic mice with AD by reducing the phosphorylation of the Tau protein (pTau) (a form that accumulates in the brain of AD patients and is the hallmark of the disease) (Green et al., 2008; Rüb et al., 2017; Nizynski et al., 2017). In the case of PD, the repletion of NAD^+^ through the use of NR prevents aging related to the loss of dopaminergic neurons and the reduction of motor function in Drosophila fly model of PD (Sidransky et al., 2009). NR improves mitochondrial function in PD neurons, increases the markers of mitochondrial biogenesis, significantly decreases the production of mtROS (mitochondrial ROS), reduces mitochondrial membrane potential and increases the levels of NAD^+^ and NADH (Schöndorf et al., 2018).

Axonal degeneration occurs in most neurodegenerative diseases (AD, PD, ALS) (Raff et al., 2002; Coleman, 2005). The increase in NAD^+^ synthesis through the use of precursors such as NR promotes axonal protection (Wang et al., 2005; Sasaki et al., 2006). Improvement of the NAD^+^ recovery pathway reverses the toxicity of primary astrocytes expressing the SOD1 mutation related to ALS, decreasing the mitochondrial production of ROS and reversing the neurotoxic effects (Harlan et al., 2016). Excitotoxicity is a process that takes place in most neurodegenerative disorders like ALS and is related to strong NAD^+^ depletion in neurons. NR protects against excitotoxicity-induced axonal generation (Vaur et al., 2017).

In Duchenne’s muscular dystrophy disease, supplementation with NR reverses progressive wear and improves resistance in skeletal muscle NAMPT in the knock out mouse model (Ryu et al., 2016).

## Pterostilbene and Neurodegenerative Diseases

Polyphenols are organic metabolites present in plants (Cory et al., 2018) and widely distributed in a variety of dietary sources (Aherne and O’Brien, 2002; Kim et al., 2009): fruits, vegetables, legumes, whole grains, seeds, nuts, extra virgin olive oil, red wine, coffee, tea, chocolate, herbs and spices (Rusconi and Conti, 2010; Fu et al., 2011; Regueiro et al., 2014; Vallverdú-Queralt et al., 2014, 2015; Talhaoui et al., 2016). Despite their zero energy contribution (Ruiz et al., 2009) they act as bioactive dietary components associated with multiple positive health effects (An-Na et al., 2014; Ganesan and Xu, 2017; Szwajgier et al., 2017; Renaud and Martinoli, 2019) due to their regulating action on metabolism, chronic diseases and cell proliferation (Tressera-Rimbau et al., 2017). Polyphenols have a chemical structure with properties that make them the compounds with the greatest antioxidant action: *o*-diphenolic group, 2-3 double bond conjugated with the 4-oxo function and hydroxyl groups (OH) in positions 3 and 5 (Ruiz et al., 2009). They have antioxidant (Hua and Rong, 2016; Tarique et al., 2016), anti-inflammatory (Oliviero et al., 2018), anticarcinogenic (Niedzwiecki et al., 2016), antiallergic (Juríková et al., 2015), antibiotic (Xie et al., 2017), and immunomodulatory (Nour et al., 2018) properties.

Polyphenols can be a useful therapeutic strategy in pathologies that present OS such as neurodegenerative diseases (Bhullar and Vasantha, 2013; Raskin et al., 2015; Reinisalo et al., 2015; Carvalho et al., 2018), cancer and cardiovascular diseases (Crozier et al., 2009; Bulotta et al., 2014; Lamuela-Raventós et al., 2014). Taking into account that nutrition is a factor that modulates processes such as cognition or progression of CNS diseases (Gustafson et al., 2015; Huhn et al., 2015), polyphenols have recently been associated with: prevention, repair of oxidative damage (Lange and Li, 2018), synaptic plasticity, neuronal signaling and autophagy (Miller and Shukitt-Hale, 2012; Poulose et al., 2014).

Among the polyphenols with important pharmaceutical activity are the stilbenes (Dvorakova and Landa, 2017), a group of non-flavonoid phytochemicals of polyphenolic structure with a 1,2-diphenylethylene core, which are produced naturally in plants via the phenylpropanoid pathway (Sirerol et al., 2016) to protect the plant from fungal infection and toxins (Akinwumi et al., 2018). There is a wide variety of forms because of a common structure to which various substituents can be added in different positions and they have an acidic and amphiphilic character (Neves et al., 2012). Although the *trans* isomer is the most common form of presentation (since it is the most stable), it can also be found in the *cis* isomer (Rivière et al., 2012). The following properties have been attributed to them: antioxidant, anti-inflammatory, neuroprotective, cardioprotective, anti-carcinogenic (Lange and Li, 2018), hypolipidemic and anti-diabetic (Voloshyna et al., 2013; Szkudelski and Szkudelska, 2015). They have great utility potential in the field of neurodegenerative diseases. They reduce the formation of amyloid plaques in the brain, decrease the production of ROS and could be of interest in other situations such as: ischemia-reperfusion injury, atherosclerosis, diabetes, cancer, obesity, platelet aggregation, blood pressure, depigmentation and cardiomyocyte and cardiac hypertrophy (Thandapilly et al., 2011; Gomez-Zorita et al., 2013; Zordoky et al., 2015; Guo et al., 2016; Akinwumi et al., 2018).

One of the most studied stilbenes has been Resveratrol (RES) (3,5,4’-trihydroxy-trans-stilbene), due to its benefits in cardiovascular health (Zordoky et al., 2015; Bonnefont-Rousselot, 2016; Dyck et al., 2019). However, recent studies focus their interest on pterostilbene (PTER) (trans-3,5-dimethoxy-4hydroxystilbene), a dimethylated natural stilbene (Albassam and Frye, 2019) with 1 hydroxyl group and 2 methoxy groups (Cichocki et al., 2008; Estrela et al., 2013; Traversi et al., 2016), which provides greater oral bioavailability, half-life, lipophilicity and higher permeability to targeted tissue (Kapetanovic et al., 2011; Yeo et al., 2013) with respect to RES (3 hydroxyl groups). In addition, PTER is less susceptible to phase II liver metabolism (Zhou et al., 2016). These particular characteristics improve its biological potential (Yeo et al., 2013) and its high bioavailability *in vivo* is an advantage over RES (Chiou et al., 2011; Carey et al., 2013; Lin et al., 2016). PTER presents a bioavailability of 80% compared to 20% for RES and plasma levels of PTER and PTER sulfate were significantly higher than plasma levels of RES and RES sulfate in a mouse model (Kapetanovic et al., 2011). After administration of PTER and RES at the same dose to male and female mice for 8 weeks, PTER reached higher concentrations in the serum and in the brain than RES (Chang et al., 2012).

The high lipophilicity of PTER allows it to easily cross the blood-brain barrier (Temsamani et al., 2015), resulting in greater neuroprotection than RES (Choo et al., 2014). In AD, PTER presents superior neuroprotection than RES (Chang et al., 2012) and is the stilbene with the highest inhibitory potential for 5-lipoxygenase (5-LOX) (Kutil et al., 2015), decreasing the levels of lipid and protein oxidation (Czpaski et al., 2012). It is a more potent anticancer and anti-inflammatory agent than RES (Chiou et al., 2011) and it distributes widely among the main target organs (brain, liver, kidney, heart and lung) for what seems to be a promising (Choo et al., 2014) and safe (Riche et al., 2013) therapeutic strategy. A study in male and female Swiss mice examined the sub-chronic toxicity and the possible adverse effects of PTER and concluded that, even with the highest dose administered, PTER was not toxic (Ruiz et al., 2009).

### Molecular Mechanisms of PTER

**FIGURE 3:**
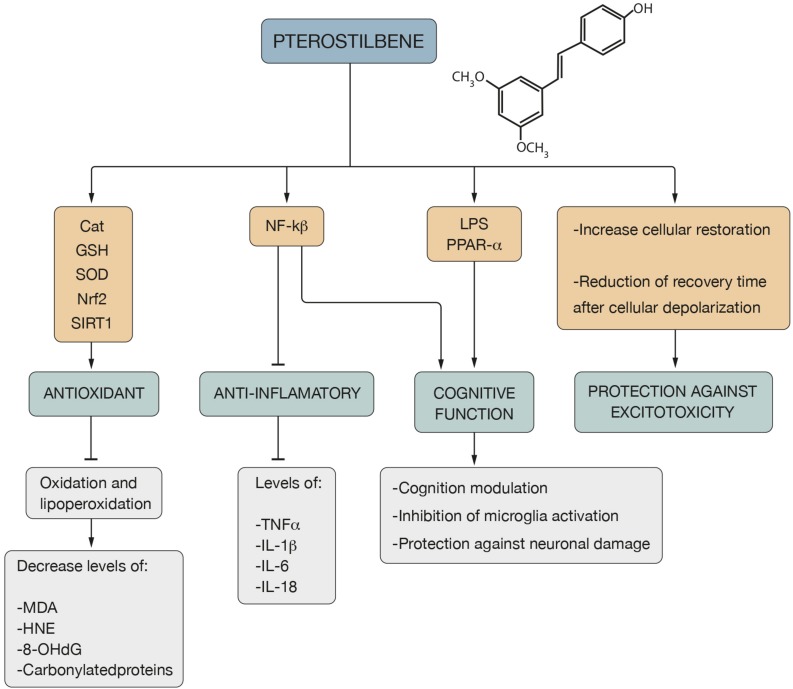
Pterostilbene properties and action mechanism. The antioxidant mechanism of Pter is associated with Cat, GSH, SOD, Nrf2 and SIRT1 pathways, which leads to an inhibition of the oxidation and lipoperoxidation processes, decreasing the levels of MDA, HNE, 8-OHdG and carbonilated proteins. Its action on NF-kß involves an anti-inflammatory effect due to the decrease in the levels of TNF-α and interleukins such as IL-1β, IL-6 and IL-18. Its role on NF-kß, LPS and PPAR-α mediates the cognitive function of Pter, which is expressed through cognition modulation, inhibition of microglia activation and protection against neuronal damage. In addition, Pter presents protection against excitotoxicity due to the increase in cellular restoration and the reduction of the recovery time after cellular depolarization.

The physiological activities of PTER include: antioxidant and anti-inflammatory activity, ability to restore intracellular calcium and cognitive function (Li Y. R. et al., 2018) (Figure 3).

#### Antioxidant Activity

PTER stands out for its antioxidant action against numerous types of free radicals: HO, H_2_O_2_ (Castellani et al., 2008; Mikstacka et al., 2010; Acharya and Ghaskadbi, 2013) and NO^∙^ (Pan et al., 2008; Zhang et al., 2012). It is able to neutralize metal-induced radicals (Rossi et al., 2013; Saw et al., 2014) and to inhibit oxidation and lipoperoxidation processes (Rimando et al., 2002) causing a decrease in carbonylated proteins and oxidation by-products: MDA, HNE and 8-OHdG (Li Y. R. et al., 2018) (Figure 3). The administration of PTER improves the antioxidant defenses of the brain by raising the levels of CAT, GSH and the activity of SOD (Naik et al., 2017) (Figure 3). In addition, diet supplemented with PTER increases the expression of SOD2 (Ding et al., 2007). Pre-treatment with PTER has a potent antioxidant function that increases SOD activity in hypoxic-ischemic brain injury in the P7 rat model (Li D. et al., 2016). Treatment with PTER reduces glutamate-induced OS injury by reducing ROS and increasing the function of SOD and GSH by activating the Nrf2 signaling pathway in neuronal cells (Wang et al., 2016), a target factor for the prevention of diseases related to aging such as neurodegenerative diseases (Bhakkiyalakshmi et al., 2016; Li Y. R. et al., 2018) (Figure 3).

The potent antioxidant mechanism of PTER is associated with SIRT1 signaling activation (Li et al., 2015; Guo et al., 2016; Liu et al., 2017) leading to the attenuation of the skeletal muscle OS injury and mitochondrial dysfunction induced by ischemia-reperfusion injury in male Sprague-Dawley rats (Cheng et al., 2016). PTER acts on Nrf2 (Saw et al., 2014; Zhang et al., 2014; Xue et al., 2017) and attenuates high glucose-induced central nervous system injury *in vitro* through the activation of Nrf2 signaling, displaying protective effects against mitochondrial dysfunction-derived OS (Yang et al., 2017).

#### Anti-inflammatory Activity

PTER has been recognized as an anti-inflammatory agent (Remsberg et al., 2008; Poulose et al., 2015; Kosuru et al., 2016) able to protect neurons against neuroinflammation due to the inhibition of ROS production (Shin et al., 2010). It acts on NF-kB and suppresses the action of various proinflammatory cytokines: TNF-α, IL-1β, IL-6 and IL-18 (Cichocki et al., 2008; Paul et al., 2009; Hou et al., 2014; El-Sayed et al., 2015) (Figure 3). PTER inhibits amyloid-β-induced neuroinflammation in microglia by inactivating the NLRP3/caspase-1 inflammasome pathway and reduces the secretion levels of IL-6, IL-1β and TNF-α, thereby attenuating the neuroinflammatory response, which would indicate that it could be a useful therapeutic strategy in neurodegenerative diseases (Li Q. et al., 2018; Seo et al., 2018).

#### Ability to Restore Intracellular Calcium

PTER is able to increase the capacity of intracellular calcium restoration by reducing recovery time after cell depolarization (Joseph et al., 2008). It protects against excitotoxicity, an aspect that would be interesting since it has been observed that the increase in intracellular ROS levels is related to damage in the cell membrane, impaired calcium homeostasis (Thibault et al., 2007; Joseph et al., 2011) and increased excitotoxicity (Rzheshevsky, 2014) (Figure 3). In the Sprague-Dawley rat model, PTER improves cholinergic transmission due to the decrease in the activity of acetylcholinesterase and increases the action of ATPases (Na^+^, K^+^, Ca^2+^, and Mg^2+^), which is indicative of the maintenance of the cell membrane potential (Naik et al., 2017).

#### Cognitive Function

After determining if PTER was effective in reversing the effects of aging in old Fischer rats, it was concluded that memory functioning was related to PTER levels in the hippocampus and that a diet supplemented with this antioxidant is effective in reversing deficits in cognitive behavior (Joseph et al., 2008). PTER attenuated lipopolysaccharide (LPS) induced learning and memory impairment, which is associated with an inhibition of the activation of microglia and therefore a protective effect against neuronal damage (Hou et al., 2014) (Figure 3). It reduces memory loss in the intracerebroventricular administered streptozotocin-induced memory decline in Sprague–Dawley rats (Naik et al., 2017) and mediates neuroprotection against oxidative toxicity via the estrogen receptor signaling pathway in human neuronal SH-SY5Y cells (Song et al., 2015).

In AD, supplementation with PTER reduces the phosphorylation of pTau (Porquet et al., 2013). The derivatives of PTER showed antioxidant and inhibitory activity against the aggregation of β-amyloid plaques, as well as cholinesterase inhibition, which would be useful in the treatment of patients with AD (Li Y. et al., 2016) and vascular dementia (Lange and Li, 2018).

PTER is a modulator of cognition and the OS due to the increased expression of peroxisome proliferator activator receptor alpha (PPAR-α) (Chang et al., 2012) (Figure 3). After comparing the effectiveness of dietary supplementation with RES and PTER in the improvement of functional deficits characteristic of AD in the SAMP8 mouse model (a model of accelerated aging that is increasingly being validated as a model of sporadic and age-related AD), it was concluded that PTER and not RES, is able to modulate the markers of cellular stress and inflammation, causing an upregulation of PPAR-α (Chang et al., 2012) and a suppression of the activation of NF-kB (Inoue et al., 2003), which is a conservative factor against the loss of cognitive function (Jeong et al., 2012; Hou et al., 2014) (Figure 3).

Due to the activation of metabolic pathways related to OS protection, inflammation, regulation of excitotoxicity and preservation of neuronal functions, PTER is a protective factor against neurodegenerative diseases (Poulose et al., 2015).

## Conclusion

OS is involved in neuroinflammation, development and progression of ALS. The complex interaction between all the factors does not allow definitive determination if OS is a primary cause or if it is a secondary effect to the propagation of damage in the nervous cells. However, a clear relationship has been established between OS, mitochondrial dysfunction and neuroinflammation, aspects that promote a “vicious cycle” that causes a decrease in the capacity of ATP biosynthesis and an increase in ROS levels.

Currently, there is no cure for ALS but the use of different antioxidant substances has been proposed as a possible therapeutic strategy, the purpose of which is to increase the body’s antioxidant defenses and maintain the redox balance. However, there are cases in which the use of these antioxidants has disadvantages or requires a higher number of studies since few results are available or these are contradictory, inconclusive or scarcely significant at the statistical level. Taking into account the pathogenic mechanisms of ALS, the new therapeutic strategies have as the main goal to activate the metabolic pathways related to the mitochondria, production of energy and increase the antioxidant defense levels.

NAD^+^ acts on energy metabolism, mitochondrial function and is key in brain metabolism, aspects involved in the pathogenesis of ALS. Restoration of NAD^+^ levels by administrating the precursor NR provides greater mitochondrial resistance against impairment of redox balance and, therefore, could play a key role in those diseases that are associated with mitochondrial dysfunction and OS.

PTER has great biological potential due to its ability to activate metabolic pathways related to protection against OS, mitochondrial dysfunction, inflammation, intracellular calcium restoration and cognitive function, thus resulting in a neuroprotective function against the pathogenic mechanisms of ALS.

In a randomized, double-blind, placebo-controlled study in humans, it was determined that repeated doses of a combination therapy with NR and PTER increased NAD^+^ levels safely and effectively (Dellinger et al., 2017). Treatment with NR and PTER is effective and safe and therefore it could be a promising therapeutic strategy in ALS, due to its action on the pathogenesis of this disease.

## Author Contributions

SC-J and MM wrote the manuscript. SC-J, MM, and ED conceived and designed the figures. CB, JR, and ED reviewed the manuscript.

## Conflict of Interest

The authors declare that the research was conducted in the absence of any commercial or financial relationships that could be construed as a potential conflict of interest.

## Abbreviations

3-NT: 3-nitrotyrosine
5-LOX: 5-lipoxygenase
7,8-DHF: 7,8-dihydroxyflavone
8-OHdG: 8-oxo-deoxyguanosine
AD: Alzheimer’s disease
ALS: amyotrophic lateral sclerosis
CAT: catalase
CNS: central nervous system
CoQ10: Co-enzyme Q10
COX: cyclooxygenase
CSF: cerebrospinal fluid
DMC: dimethoxy curcumin
EGCG: epigallocatechin gallate
ETC: electron transport chain
F2-IsoPS: F2-isoprostane
fALS: familial amyotrophic lateral sclerosis
GPx: Glutathione peroxidase
GR: Glutathione reductase
GSH: Reduced glutathione
GSK-3ß: Glycogen synthase kinase 3 beta
GSSG: Glutathione disulfide
H_2_O_2_: Hydrogen peroxide
HD: Huntington’s disease
HNE: 4-hydroxy-2-nonenal
IDH2: Isocitrate dehydrogenase
IL-18: Interleukin-18
IL-1ß: Interleukin 1ß
IL-6: Interleukin-6
IL-8: Interleukin-8
iNOS: nitric oxide synthase
LDL: low-density lipoprotein
LMN: lower motor neurons
LOX: lipoxygenase
LPS: lipopolysaccharide
MDA: malondialdehyde
MPO: myeloperoxidase
MS: multiple sclerosis
mtDNA: mitochondrial DNA
mtROS: mitochondrial reactive oxygen species
NA: nicotinic acid
NAD^+^: nicotinamide adenine dinucleotide
NADPH: nicotinamide adenine dinucleotide phosphate
NAM: nicotinamide
NAMPT: nicotinamide phosphoribosyltransferase
NF-kB: nuclear factor kappa-light-chain-enhancer of activated B cells
NMN: nicotinamide mononucleotide
NO: nitric oxide
NO^–^: nitroxyl anion
NO^+^: nitrosonium cation
NO ^∙^: nitric oxide radical
NOS: nitric oxide synthase
NR: nicotinamide riboside
Nrf2: nuclear factor erythroid 2-related factor
NRK1: nicotinamide riboside kinase 1
NRK2: nicotinamide riboside kinase 2
NRKs: nicotinamide riboside kinases
O_2_: singlet oxygen
O${}_{2}^{∙-}$: superoxide anion radical
OH: hydroxyl group
OH ^∙^: hydroxyl radical
ONOO^–^: peroxynitrite anion
OS: oxidative stress
PARPs: poly(ADP-ribose) polymerase
PD: Parkinson’s disease
PGC-1 α: Peroxisome proliferator-activated receptor gamma coactivator 1-alpha
PNP: purine nucleoside phosphorylase
PPAR- α: peroxisome proliferator activator receptor alpha
pTau: Tau protein
PTER: Pterostilbene (trans-3,5-dimethoxy-4 hydroxystilbene)
PUFAs: polyunsaturated fatty acids
Q3BDG: Quercetin 3-ß-D-glucoside
RCS: reactive species of copper
RES: Resveratrol (3,5,4 ′ -trihydroxy-*trans*-stilbene)
RIS: reactive iron species
RNA: ribonucleic acid
RNS: reactive nitrogen species
RONS: reactive oxygen and nitrogen species
ROS: reactive oxygen species
SA: spinocerebellar ataxia
sALS: sporadic amyotrophic lateral sclerosis
SIRT: sirtuin
SIRT1: sirtuin 1
SIRT3: sirtuin 3
SOD: superoxide dismutase
SOD1: superoxide dismutase 1
SOD2: superoxide dismutase 2
TAS: total antioxidant status
TBARS: thiobarbituric acid reactive species
TNF- α: tumor necrosis factor alpha
UMN: upper motor neurons
XO: xanthine oxidase.
